# CD20 × CD3 Bispecific Antibodies in B-Cell Non-Hodgkin Lymphomas: Current Evidence, Therapeutic Integration, and Future Directions

**DOI:** 10.3390/medicina62061056

**Published:** 2026-05-29

**Authors:** Polyxeni Giamaiou, Rodanthi Fioretzaki, Theodoros P. Vassilakopoulos, Maria Dimou

**Affiliations:** Department of Haematology and Bone Marrow Transplantation Unit, National and Kapodistrian University of Athens, Laikon General Hospital, 11527 Athens, Greece; polyxenigiamaioukm@gmail.com (P.G.); rodanthifioretzaki@gmail.com (R.F.); theopvass@hotmail.com (T.P.V.)

**Keywords:** bispecific antibodies, CD20 × CD3, B-cell non-Hodgkin lymphoma, diffuse large B-cell lymphoma, follicular lymphoma, CRS, ICANS

## Abstract

*Background and Objectives*: Relapsed or refractory (R/R) B-cell non-Hodgkin lymphomas (B-NHL) remain associated with poor outcomes despite advances in chemoimmunotherapy and chimeric antigen receptor (CAR) T-cell therapy. Many patients are ineligible for or relapse after cellular therapies, highlighting the need for effective off-the-shelf immunotherapeutic approaches. CD20 × CD3 bispecific antibodies (BsAbs) redirect endogenous T cells against malignant B cells and have emerged as a promising therapeutic class in B-NHL. To summarize current clinical evidence regarding mosunetuzumab, glofitamab, epcoritamab, and odronextamab in B-NHL, focusing on efficacy, safety, and emerging therapeutic applications. *Materials and Methods*: A structured review of published phase I–III clinical trials evaluating the four currently approved CD20 × CD3 BsAbs in B-NHL was conducted. Efficacy outcomes, durability of response, and safety data were assessed across indolent and aggressive lymphoma subtypes. *Results*: CD20 × CD3 BsAbs demonstrated substantial and durable clinical activity in heavily pretreated B-NHL, including patients with prior CAR T-cell exposure. Mosunetuzumab showed high response rates and durable remissions in follicular lymphoma (FL), while glofitamab demonstrated significant efficacy in aggressive lymphomas, particularly diffuse large B-cell lymphoma (DLBCL). Epcoritamab exhibited consistent activity across lymphoma subtypes with favorable tolerability supported by subcutaneous administration and step-up dosing. Odronextamab also demonstrated clinically meaningful responses in both FL and DLBCL, including high-risk populations. Across studies, cytokine release syndrome (CRS) was the most common adverse event, predominantly low grade and manageable with established mitigation strategies. Immune effector cell-associated neurotoxicity syndrome (ICANS) was uncommon. Infections and hematologic toxicities, particularly neutropenia, represented clinically relevant adverse events across all treatment programs, highlighting the need for special supportive care. *Conclusions*: CD20 × CD3 BsAbs represent a major therapeutic advancement in R/R B-NHL, combining high clinical activity, manageable toxicity, and off-the-shelf availability. Their expanding integration into earlier treatment settings and combination strategies is expected to further reshape the therapeutic landscape of B-NHL.

## 1. Introduction

B-NHLs comprise a diverse spectrum of lymphoid cancers, varying significantly in their biological behavior and clinical progression [1]. Historically, the standard frontline treatment for B-NHL has relied on combining anti-CD20 monoclonal antibodies, such as rituximab, with cytotoxic chemotherapy regimens (e.g., CHOP or bendamustine) [2]. While these combinations have drastically increased survival rates and enabled long-term remission for many, a substantial number of patients—particularly those with high-risk features or aggressive histologies—still face disease recurrence [3]. For patients with R/R DLBCL, the established protocol was long centered on salvage chemoimmunotherapy followed by autologous stem cell transplantation (ASCT) [4]. In recent years, this paradigm has been challenged by the introduction of CD19-directed chimeric antigen receptor (CAR) T-cell therapies. CAR-T therapy has demonstrated the ability to produce profound and lasting responses in patient groups that previously had very limited clinical options [5,6,7]. Despite the success of CAR-T cells, their widespread clinical utility is hindered by several significant obstacles. The personalized manufacturing of CAR-T cells is technically demanding and time-intensive. Specific side effects, such as CRS, and restricted availability to specialized centers limit the number of eligible patients [8]. Outcomes remain exceptionally poor for patients who relapse after CAR T-cells [9]. In addition, the management of treatment-related toxicities and the identification of optimal sequencing strategies remain ongoing clinical challenges in this setting.

In indolent lymphomas, particularly FL, the disease course is characterized by repeated relapses and progressively shorter remissions, with a subset of patients exhibiting high-risk features such as early progression within 24 months (POD24) [10]. Although most patients initially respond well to anti-CD20-based chemoimmunotherapy, FL remains incurable, and successive lines of treatment are associated with increasing immunosuppression and declining tolerability [11]. CD19-directed CAR T-cell therapy has demonstrated high response rates and durable remissions in heavily pretreated FL, establishing a highly effective treatment option in the R/R FL setting [12,13]. However, its use is limited by manufacturing complexity, treatment-related toxicities, and restricted accessibility [8].

In this context, T-cell-redirecting BsAbs have emerged as a promising off-the-shelf alternative, offering the potential for deep and durable responses without the logistical constraints associated with cellular therapies, and are now established treatment options in the R/R setting [14,15]. Although they share a common mechanism of action, emerging clinical data suggest differences in safety profiles, particularly with regard to CRS and infection risk, which may influence their clinical use. At the same time, despite substantial improvements in frontline therapy, a significant proportion of patients with DLBCL either fail to achieve durable remission or relapse early after standard chemoimmunotherapy, while in FL long-term disease control remains elusive, particularly in high-risk subgroups [3,10]. This has led to the evaluation of BsAbs in earlier lines of therapy, including the frontline setting, in both DLBCL and FL [16].

This review aims to provide a concise overview of the four currently approved CD20 × CD3 BsAbs in B-NHLs, namely mosunetuzumab, glofitamab, epcoritamab, and odronextamab (Table 1), focusing on their structural characteristics, mechanisms of action, adverse event profile and emerging clinical role across different lines of therapy, with particular emphasis on comparative clinical outcomes and safety profile, both within their approved indications and across other B-NHL subtypes and treatment settings.

## 2. Structure and Mechanism of Action of Bispecific Antibodies

BsAbs are engineered antibodies that bind two different targets at the same time, typically CD3 on T cells and CD20 on B cells, enabling direct T-cell-mediated cytotoxicity [24]. The currently available CD20 × CD3 BsAbs are all IgG-like molecules with an Fc region, which provides improved stability and longer systemic exposure compared to earlier fragment-based constructs. In most cases, the Fc domain is modified to reduce Fcγ receptor binding and limit non-specific immune activation [24,25]. Although these agents share the same overall design principle, there are relevant structural differences between them that may influence their biological behavior [15,25,26].

Mosunetuzumab, epcoritamab and odronextamab are CD20 × CD3 BsAbs with IgG-like architectures. All three engage CD20 and CD3 in a functionally monovalent (1:1) manner; however, they differ in their molecular engineering platforms (CrossMAb for mosunetuzumab, DuoBody for epcoritamab, and an IgG4-based heterodimeric platform for odronextamab) and domain arrangement, which are designed to ensure correct heavy/light chain pairing and structural stability. CrossMAb-based designs use domain crossover strategies to minimize chain mispairing, whereas DuoBody technology relies on controlled Fab-arm exchange between parental antibodies to generate a stable bispecific molecule. These structural differences, although not always evident in simplified schematics, may influence binding geometry, pharmacokinetic behavior and other translational properties [15,25,26].

Glofitamab, in contrast, has a distinct 2:1 configuration, with two binding sites for CD20 and one for CD3. This bivalent engagement of CD20 increases avidity for the target cell and may enhance activity in settings of lower antigen density, although the clinical implications of this design remain to be fully elucidated [15,26].

Beyond valency, additional design features further differentiate these molecules. Epcoritamab uses a subcutaneous formulation, which results in slower absorption and more gradual systemic exposure, while the other agents are administered intravenously. Subtle differences in Fc engineering and molecular architecture also contribute to variability in half-life, cytokine release patterns, and overall tolerability, although these effects are not solely determined by structure [21,22,26].

**Figure 1:**
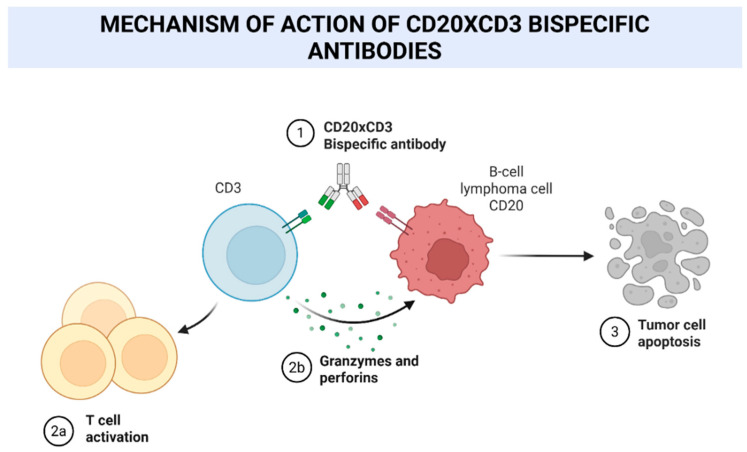
Mechanism of action of CD20 × CD3 bispecific antibodies. Created in BioRender. Giamaiou, P. (2026). Available online: https://BioRender.com/ux3ki7x (accessed on 30 March 2026). (1) The bispecific antibody simultaneously binds CD20 on malignant B-cell lymphoma cells and CD3 on T lymphocytes. (2a) This interaction results in T-cell activation. (2b) Activated T cells release cytotoxic molecules, including perforins and granzymes. (3) These mechanisms ultimately induce apoptosis of the tumor cell.

Despite these differences, the mechanism of action is shared across all agents. By simultaneously engaging CD3-positive T cells and CD20-positive B cells, BsAbs induce the formation of an artificial immune synapse, leading to T-cell activation independently of MHC-mediated antigen presentation. Activated T cells then mediate cytotoxicity through perforin and granzyme release and produce pro-inflammatory cytokines (Figure 1). Importantly, a single T cell can eliminate multiple tumor cells sequentially, contributing to the potency of this class [15,27].

## 3. Pivotal Trials and Ongoing Clinical Development of Mosunetuzumab

Mosunetuzumab represents a landmark in immunotherapy as the first BsAb to achieve regulatory authorization, specifically for adult patients with R/R FL following at least two prior lines of systemic therapy, which currently remains its only approved indication [17,18]. The approval of mosunetuzumab was based on the pivotal phase II GO29781 study, a single-arm trial evaluating patients with heavily pretreated disease, including prior exposure to alkylating agents and anti-CD20 monoclonal antibodies [28].

Its clinical application utilizes a step-up dosing strategy designed to minimize the risk of CRS during the initial treatment cycles. This approach has been consistently associated with a predictable CRS profile characterized by early onset, predominantly during cycle 1, and largely low-grade events. Long-term follow-up at a median of 37.4 months demonstrated an objective response rate (ORR) of 78% and a complete response (CR) rate of 60%, exceeding historical benchmarks for third-line FL therapy. The durability of response was notable, with a median duration of response (DOR) of 35.9 months, while the median duration of CR had not been reached at the time of analysis. Overall survival (OS) was also favorable, with a 36-month OS rate of 82.4% [28,29].

Safety data showed that CRS occurred in 44.4% of patients, but was predominantly low-grade, with only 2.2% experiencing grade ≥ 3 events. No cases of ICANS were reported [29]. Importantly, CRS events were largely confined to the first cycle and decreased substantially with subsequent dosing, supporting the effectiveness of the step-up strategy. Beyond CRS, the safety profile is characterized by manageable hematologic toxicity, with neutropenia representing the most common grade ≥ 3 event, typically without a corresponding increase in febrile neutropenia. Infections represent a clinically relevant component of the safety profile. Although overall infection rates were not consistently reported across studies, specific events such as pneumonia (4.6%, grade ≥ 3: 2.5%) and urinary tract infections (7.6%, grade ≥ 3: 2.5%) were observed, whereas severe opportunistic infections appeared uncommon. In addition, retreatment in a small subset of patients suggested maintained sensitivity to the drug, with a 40% CR rate and a safety profile comparable to initial exposure [29].

Within this clinical framework, mosunetuzumab is administered intravenously on a step-up dosing schedule during the first cycle to mitigate the risk of CRS. Patients receive 1 mg on day 1 and 2 mg on day 8 of cycle 1, followed by 60 mg on day 15. In subsequent cycles, the drug is administered at 60 mg in cycle 2 and 30 mg from cycle 3 onwards, in 21-day cycles. Treatment is given for a fixed duration of up to 8 cycles in patients achieving CR, or up to 17 cycles in those with partial response or stable disease. Premedication and close monitoring are recommended during the initial step-up doses, particularly in cycle 1. However, hospitalization is not required, and treatment can be delivered in the outpatient setting in experienced centers [17,18].

In the phase Ib/II GO40516 study, intravenously administered mosunetuzumab in combination with polatuzumab vedotin was evaluated in patients with R/R large B-cell lymphoma (LBCL). The study demonstrated encouraging activity with a manageable safety profile and supported the further development of mosunetuzumab-based combination strategies [30]. This provided the rationale for the subsequent randomized phase III SUNMO trial, in which subcutaneous mosunetuzumab plus polatuzumab vedotin was compared with rituximab, gemcitabine, and oxaliplatin in transplant-ineligible patients with R/R LBCL. The SUNMO study demonstrated a clear clinical benefit for the mosunetuzumab-based regimen, with improved progression-free survival (PFS), higher CRRs, and high ORRs, supporting its role as a chemotherapy-free option in this setting. Adverse events extended beyond CRS and ICANS and included infections, neutropenia, and peripheral neuropathy, reflecting the contribution of polatuzumab-based therapy and highlighting the need for careful toxicity monitoring in combination regimens. Notably, the mosunetuzumab-containing regimen showed a favorable toxicity profile compared with chemoimmunotherapy, with lower rates of thrombocytopenia and peripheral neuropathy, while maintaining comparable rates of serious adverse events. The infectious profile was predominantly driven by viral respiratory events, with COVID-19 being the most commonly reported infection (15% vs. 3.1%), whereas pneumonia occurred less frequently (5.2% vs. 0.0%) [31].

In the frontline setting of DLBCL, mosunetuzumab has also been combined with standard chemotherapy. A phase II study of mosunetuzumab plus CHOP demonstrated manageable safety and encouraging activity in previously untreated patients. CRS remained predominantly low-grade in this setting, and no consistent signal of clinically relevant neurotoxicity was observed. Hematologic toxicity was mainly driven by neutropenia (15%; grade ≥ 3: 13%), with febrile neutropenia being rare (1.9%). Infections occurred in 22% of patients, with a considerable proportion being grade ≥ 3 (21%). Severe infections were predominantly COVID-19-related pneumonia, underscoring the need for careful infection monitoring in this setting [32].

This approach was further explored in a study comparing Pola-M-CHP with Pola-R-CHP, where response rates were similar between the two arms, suggesting that mosunetuzumab can be incorporated into frontline regimens, although without clear clinical benefit over rituximab in this setting. Although neurologic adverse events were reported, none were ultimately classified as ICANS after clinical adjudication, further supporting a favorable neurologic safety profile. In the Pola-M-CHP arm, infections were reported in 10.5% of patients (grade ≥ 3: 7.9%), although detailed characterization of infection types was limited [33].

In parallel, the phase II platform MorningSun trial is exploring subcutaneous mosunetuzumab in selected elderly/unfit populations, to enable a more convenient outpatient administration. In the cohort of untreated DLBCL patients, interim results of efficacy and safety were promising. Subcutaneous administration appears to further reduce the incidence and severity of CRS, while maintaining overall tolerability [34].

In FL, one of the most important ongoing studies is the phase III CELESTIMO trial, which compares mosunetuzumab plus lenalidomide with rituximab plus lenalidomide (R2) in patients who have received at least one prior line of therapy. Preliminary results from the US extension cohort (Arm C) of the phase III CELESTIMO trial presented at ASH 2025 demonstrated high response rates with mosunetuzumab plus lenalidomide in patients with R/R FL, with an ORR of 96.3% and a CR rate of 87.0%, although these findings derive from a non-randomized cohort and comparative data versus the control arm are not yet available The addition of lenalidomide did not appear to substantially alter the safety profile, with CRS remaining mostly low-grade and cytopenias representing the main grade ≥ 3 toxicities. Infections were mainly respiratory and viral, with COVID-19 being the most frequently reported event, followed by sinusitis and upper respiratory tract infections [35].

Mosunetuzumab is also being evaluated in the frontline treatment of FL. In the MorningSun study, subcutaneous mosunetuzumab demonstrated high response rates and encouraging PFS in previously untreated high tumor burden FL cohort, with a manageable safety profile. Notably, responses were maintained over time, and the optional maintenance cohort suggested sustained disease control without additional safety signals. Injection-site reactions were common with subcutaneous administration but were predominantly low-grade and self-limited, while CRS events remained low-grade and fully reversible. Infections were predominantly respiratory, including upper respiratory tract infections (21.8%), COVID-19 (17.8%), sinusitis (13.9%), and pneumonia (11.9%) [36]. The phase III MorningLyte trial sponsored by LYSA (The Lymphoma Academic Research organization) is currently recruiting patients and compares mosunetuzumab plus lenalidomide with standard chemoimmunotherapy in untreated patients, investigating a chemotherapy-free alternative [37].

Several smaller or earlier-phase studies are also ongoing. In patients with early-relapsing FL, the phase II MERLIN/NLG-FL6 study is evaluating subcutaneous mosunetuzumab in a high-risk population with POD24 [38]. In untreated FL, mosunetuzumab is being combined with tazemetostat, based on the idea that epigenetic modulation may enhance immune responses [39].

Finally, mosunetuzumab is also being studied in biologically aggressive disease. A phase Ib/II trial is evaluating its combination with dose-adjusted EPOCH in patients with newly diagnosed high-grade B-cell lymphoma or DLBCL with MYC rearrangements, including double-hit lymphomas. Although still early, this reflects the effort to move BsAbs into high-risk frontline settings [40]. Across these diverse settings, mosunetuzumab maintains a predictable and manageable safety profile, with a predominantly respiratory-driven infectious pattern and no evidence of cumulative or delayed toxicity. The pivotal and ongoing clinical trials of mosunetuzumab in B-cell lymphomas across different disease settings are summarized in (Table 2).

## 4. Pivotal Trials and Ongoing Clinical Development of Glofitamab

The clinical utility of glofitamab has been established through the pivotal NP30179 phase I/II study (NCT03075696) [53,54,55,56]. This trial evaluated glofitamab in patients with R/R B-NHL, including mantle cell lymphoma (MCL) and large B-cell lymphoma (LBCL), incorporating obinutuzumab pretreatment to mitigate CRS. In patients with relapsed or refractory MCL, glofitamab demonstrated high activity, achieving a CR rate of 78.3% and an ORR of 85%, including in those previously exposed to Bruton tyrosine kinase inhibitors. The safety profile in this cohort was characterized by frequent CRS and hematologic toxicity, with neutropenia representing the most common grade ≥ 3 event. Infections were common and included COVID-19, COVID-19-related pneumonia, and bacterial pneumonia, while fatal infection-related adverse events were reported, including COVID-19-related events, septic shock, and pneumonia [54].

In a broader cohort of heavily pretreated B-NHL, glofitamab induced ORR of approximately 54–66% and CR rates up to 57%, with durable remissions extending beyond two years in a substantial proportion of patients [55,56]. Although CRS was common, events were predominantly low grade and manageable, particularly with the implementation of step-up dosing and obinutuzumab pretreatment [55,56,57]. Importantly, patient-reported outcomes demonstrated maintenance or improvement in health-related quality of life and lymphoma-associated symptoms during treatment. Neurologic adverse events consistent with ICANS were infrequent and included occasional grade ≥ 3 events, remaining generally reversible. Infections were frequent and included COVID-19 and COVID-19-related pneumonia as the most common events, with sepsis representing an additional cause of severe infection. Despite the high incidence of neutropenia, febrile neutropenia remained uncommon, and only low rates of additional complications such as tumor lysis syndrome were observed. Importantly, patient-reported outcomes demonstrated maintenance or improvement in health-related quality of life and lymphoma-associated symptoms during treatment [58].

Evidence supporting the activity of glofitamab in patients with R/R DLBCL following CAR T-cell therapy derives from both subgroup analyses of NP30179 and dedicated prospective studies. Subgroup analyses suggested comparable response rates in patients previously treated with CAR T-cell therapy and the overall study population, with durable responses observed [55]. These findings are further supported by a prospective phase II LYSA study, which reported a CR rate of 45.7%, an ORR of 76.1%, and a median OS of 14.7 months in patients with DLBCL after CAR T-cell failure, without an increased incidence of severe CRS or ICANS. ICANS was observed in a minority of patients, including grade ≥ 3 events, but remained infrequent and typically reversible [59].

Overall, NP30179 established glofitamab as an effective fixed-duration bispecific antibody therapy with a manageable safety profile in R/R B-NHL, supporting its regulatory approval and subsequent development in combination regimens. Across cohorts, neutropenia was the most common grade ≥ 3 adverse event, while febrile neutropenia remained uncommon. Infections constituted a clinically relevant component of the safety profile, predominantly involving COVID-19 and COVID-19-related pneumonia, with additional cases of bacterial pneumonia and sepsis, including septic shock, reported among severe and fatal events [55,56].

To further reduce the risk of CRS, NP30179 employed a step-up dosing strategy combined with obinutuzumab pretreatment [57]. Patients received obinutuzumab on day 1 of cycle 1, followed by escalating doses of glofitamab (2.5 mg on day 8 and 10 mg on day 15), before administration of the full target dose in subsequent cycles. This approach enabled controlled immune activation while maintaining high efficacy and limiting severe CRS events [55,57].

The phase III STARGLO trial established glofitamab in combination with gemcitabine and oxaliplatin (Glofit-GemOx) as a highly effective treatment option for patients with relapsed or refractory DLBCL who are ineligible for autologous stem cell transplantation [59,60,61,62]. In this randomized study, Glofit-GemOx significantly improved OS compared with R-GemOx, with a median OS of 25.5 months versus 12.5 months, respectively [62]. The regimen also demonstrated improved response rates and PFS, with the greatest benefit observed in patients treated in earlier lines of therapy [61,62]. The safety profile remained consistent with previous studies, with predominantly low-grade CRS and no evidence of cumulative toxicity [61]. Adverse events of special interest included CRS, neurologic adverse events, serious infections, febrile neutropenia, and tumor flares. CRS occurred predominantly during the initial step-up dosing phase and was mainly low grade. ICANS events were uncommon, mostly low grade with rare grade ≥ 3 events, and consistently occurred concurrently with CRS, resolving with its management. A higher incidence of CRS was observed in the glofitamab arm compared with the chemotherapy control arm. Infections were driven by COVID-19, which represented the most frequently reported serious infection and the leading cause of fatal adverse events in the glofitamab arm, while no COVID-19-related deaths were observed in the comparator arm. COVID-19-related events also contributed to treatment discontinuation in a proportion of patients [62].

Combination strategies incorporating glofitamab and polatuzumab vedotin have demonstrated promising efficacy in R/R LBCL. In the phase Ib/II study (NCT03533283), this combination achieved an ORR of approximately 80% and a CR rate of 59.7%, with a median PFS of 12.3 months and durable responses extending beyond two years [63,64,65,66]. Subgroup analyses indicate consistent efficacy across treatment lines. The safety profile was characterized by frequent CRS, which occurred early during treatment and was predominantly low grade with predictable onset and resolution. Infections were common and included SARS-CoV-2 infection and pneumonia as the most frequent events, with severe infections primarily driven by COVID-19-related events and pneumonia. Fatal adverse events were mainly infection-related, including COVID-19-associated events, sepsis, and rare cases of progressive multifocal leukoencephalopathy. Hematologic toxicity was notable, with neutropenia representing the most common high-grade event, alongside anemia and thrombocytopenia. ICANS events were infrequent, low grade, and reversible, while peripheral neuropathy was observed but limited to low-grade events [64,65,66].

Given these encouraging results, the development of glofitamab is expanding into frontline therapy. Several ongoing trials are evaluating its integration into standard chemoimmunotherapy backbones [67,68,69]. In the phase II COALITION study, the addition of glofitamab to R-CHOP or polatuzumab-based regimens in younger patients with high-risk LBCL resulted in an ORR of 100% and CR rates approaching 98%, with only low-grade CRS events reported. Across early-phase and optimization studies, including modified dosing strategies, no increase in grade ≥ 3 CRS or neurotoxicity was observed [68].

In parallel, biomarker-driven strategies are being explored to optimize patient selection. A phase II study using circulating tumor DNA (ctDNA) to identify patients with suboptimal early response demonstrated that the addition of glofitamab to R-CHOP achieved CR rates of approximately 80% in a response-adapted approach, highlighting the potential for personalized treatment strategies [70].

Building on these findings, the ongoing phase III SKYGLO trial is evaluating glofitamab in combination with polatuzumab-based chemoimmunotherapy in previously untreated LBCL, intending to further improve long-term outcomes [67]. Overall, glofitamab is associated with early-onset CRS, infrequent but present neurotoxicity, and a consistent infectious pattern predominantly involving COVID-19 and pneumonia, with sepsis representing an additional cause of severe infection. Compared with other CD20 × CD3 BsAbs, glofitamab demonstrates a more pronounced early CRS profile with predictable kinetics while maintaining a comparable overall safety profile, with infections largely driven by respiratory viral events. The primary clinical trials evaluating glofitamab across these disease settings are summarized in (Table 3).

## 5. Pivotal Trials and Ongoing Clinical Development of Epcoritamab

Epcoritamab has one of the broadest clinical development programs among CD20 × CD3 BsAbs in B-NHL. Its development has been built around two major platform studies, EPCORE NHL-1 and EPCORE NHL-2 [76,77], and has subsequently expanded into dedicated phase III trials in both DLBCL and FL. Epcoritamab is currently approved by both the FDA and EMA as monotherapy for adults with R/R DLBCL and FL after at least two prior lines of systemic therapy; in addition, the FDA has approved epcoritamab in combination with rituximab and lenalidomide for R/R FL [21,22].

The EPCORE NHL-1 study (NCT03625037) is a multicenter, open-label, phase I/II trial that evaluated subcutaneous epcoritamab monotherapy in adult patients with R/R CD20-positive B-NHL [76]. In the dose-escalation phase, epcoritamab was explored across doses ranging from 0.0128 mg to 60 mg using step-up schedules designed to reduce CRS. No dose-limiting toxicities were observed and the maximum tolerated dose was not reached; based on the overall safety and exposure profile, 48 mg was selected as the recommended phase II dose (RP2D). In this early cohort, tolerability was acceptable, with predominantly low-grade CRS, pyrexia, and injection-site reactions, while clinically meaningful activity was already evident in both DLBCL and FL [78].

The pivotal expansion cohort of EPCORE NHL-1 established epcoritamab monotherapy as an active treatment option in R/R large B-cell lymphoma (LBCL) after at least two prior lines of therapy. In the primary analysis, subcutaneous epcoritamab achieved an ORR of 63.1% and a CR rate of 38.9%, with a median DoR of 12.0 months and a median duration of CR (DoCR) of 20.8 months [79]. With longer follow-up, responses remained durable, supporting the long-term clinical value of this approach in heavily pretreated LBCL. In the 3-year EPCORE NHL-1 subgroup analysis, epcoritamab demonstrated sustained activity in patients with prior CAR T-cell therapy, with ORR 41.9–58.3% and CR 22.6–50.0% depending on timing of relapse, while median DoCR was not reached Across cohorts, CRS was frequent but largely low grade and occurred early during treatment, whereas ICANS was uncommon and generally reversible [80].

In R/R FL, the phase II cohort of the EPCORE NHL-1 study provided the basis for the regulatory approval of epcoritamab [21,22]. A total of 128 patients received subcutaneous epcoritamab at the RP2D of 48 mg, with a median of three prior lines of therapy and a high proportion of double-refractory disease. Epcoritamab achieved an ORR of 82% and a CR rate of 63%, with consistent activity across high-risk subgroups, including patients with progression of disease within 24 months (POD24) and high FLIPI scores. In a dedicated cycle 1 optimization cohort, an additional 3 mg step-up dose was introduced on day 15 before the first full 48 mg dose on day 22; response rates were maintained (ORR 86%, CR 64%), while the incidence of CRS decreased to 49%, with no grade ≥ 3 events. Overall, CRS was reported in 66% of patients and was mainly grade 1–2, while ICANS occurred in 6% of patients, all low grade. Injection-site reactions and pyrexia were common, whereas hematologic toxicity remained limited and manageable. These findings confirm both the high efficacy and the favorable tolerability of epcoritamab in heavily pretreated FL [81].

Within this clinical framework, epcoritamab is administered subcutaneously using step-up dosing during cycle 1 to mitigate the risk of CRS. In LBCL, the approved schedule consists of a two-step-up regimen with 0.16 mg on day 1, 0.8 mg on day 8, and the first full 48 mg dose on day 15, whereas in FL a three-step-up regimen is used, with 0.16 mg on day 1, 0.8 mg on day 8, 3 mg on day 15, and the first full 48 mg dose on day 22. Premedication with corticosteroids is recommended during cycle 1, administered prior to each weekly epcoritamab dose and continued for three consecutive days after each administration to reduce the risk of CRS. After completion of the step-up phase, epcoritamab is given weekly during cycles 1–3, every two weeks during cycles 4–9, and every four weeks thereafter [78,81].

The EPCORE NHL-2 study (NCT04663347) is a multicohort phase I/II platform trial evaluating epcoritamab across ten predefined combination cohorts spanning both frontline and R/R settings in DLBCL and FL [77,82]. Among these, the most mature clinical data derive from the R/R DLBCL cohorts. In transplant-ineligible patients, the combination with gemcitabine and oxaliplatin (GemOx) demonstrated robust activity, with ORR of 85% and CR of 61%, alongside durable responses [83]. The tolerability profile was consistent with monotherapy, characterized by predominantly low-grade CRS and infrequent ICANS, without new safety signals. In transplant-eligible populations, epcoritamab combined with R-DHAX/C and R-ICE has also shown high CR rates, supporting its integration into salvage regimens prior to ASCT [84]. In untreated DLBCL, combinations with R-CHOP and R-mini-CHOP for elderly patients have also yielded very high response rates [83,84].

In FL, the EPCORE NHL-2 platform has demonstrated consistent activity across both relapsed and frontline settings. In the R/R setting, epcoritamab combined with lenalidomide and rituximab (R^2^) achieved high response rates, including deep remissions [77]. In the frontline setting, lenalidomide-based combinations have been associated with durable responses and encouraging long-term outcomes. Epcoritamab plus bendamustine and rituximab (BR) in untreated FL has also shown high CR rates in early analyses [85]. In addition, a dedicated epcoritamab maintenance cohort is evaluating patients who achieved response after prior therapy, with preliminary data suggesting sustained disease control. Across FL cohorts, treatment was well tolerated, with low rates of grade ≥ 3 CRS and ICANS and no unexpected safety signals [77,82,85].

Several phase III trials are currently shaping the role of epcoritamab across both indolent and aggressive B-cell lymphomas.

The most mature evidence to date comes from the EPCORE FL-1 trial (NCT05409066), a randomized phase III study evaluating fixed-duration epcoritamab in combination with R^2^ versus R^2^ in patients with R/R FL after at least one prior line of therapy [86,87]. The study demonstrated a clear efficacy benefit for the epcoritamab-based regimen, with ORR of approximately 95% compared with 79% in the control arm and a marked improvement in PFS (HR~0.2), establishing this chemo-free combination as a new standard option in this setting. Responses were deep and consistent across high-risk subgroups, while CRS remained manageable within the step-up dosing strategy. However, higher rates of grade ≥ 3 and serious adverse events were observed with epcoritamab plus R^2^ compared with R^2^ alone, primarily driven by hematologic toxicity and infections. Neutropenia was the most frequent high-grade adverse event, with increased rates of febrile neutropenia and thrombocytopenia also observed in the combination arm. Infections were common and included COVID-19, upper respiratory tract infections, and pneumonia as the most frequent events, with a higher incidence of grade ≥ 3 and opportunistic infections, including cytomegalovirus and herpes virus infections. Despite this, fatal infections were infrequent and comparable between treatment groups. Treatment discontinuation occurred more frequently in the epcoritamab combination arm and was most commonly driven by infections, cytopenias, and rash. CRS remained largely low grade, and its incidence decreased with optimized step-up dosing strategies [87].

In aggressive lymphoma, the phase III EPCORE DLBCL-1 trial (NCT04628494) is evaluating epcoritamab monotherapy versus investigator’s choice (R-GemOx or BR) in patients with R/R DLBCL who are ineligible for ASCT [88]. In January 2026, AbbVie announced topline results showing a statistically significant improvement in PFS with epcoritamab (HR 0.74, 95% CI 0.60–0.92), together with improvements in CR, DoR, and time to next treatment (TTNT), whereas OS was not significantly different (HR 0.96, 95% CI 0.77–1.20). The safety profile was consistent with prior studies, with CRS occurring predominantly during the first treatment cycle and remaining largely low grade. Neurotoxicity consistent with ICANS was infrequent and manageable. The most common adverse events included fatigue and pyrexia, in line with the established safety profile of epcoritamab [89].

In the frontline setting, epcoritamab is currently being evaluated in the phase III EPCORE DLBCL-2 trial (NCT05578976), which compares subcutaneous epcoritamab plus R-CHOP with standard R-CHOP in previously untreated DLBCL [90]. In parallel, the phase III EPCORE DLBCL-4 study (NCT06508658) is assessing a chemotherapy-free approach in the relapsed setting, comparing epcoritamab plus lenalidomide with R-GemOx in patients who are not candidates for cellular therapies and ASCT [91]. Together, these ongoing trials extend the clinical development of epcoritamab beyond monotherapy and are expected to further define its role across both frontline and relapsed disease settings [78,89,90,91].

Overall, epcoritamab is characterized by a favorable safety profile, with CRS typically low grade, infrequent neurotoxicity, and a manageable pattern of infections, primarily involving respiratory tract infections including COVID-19 and pneumonia, without unexpected safety signals across treatment settings. The key clinical studies of epcoritamab across different disease settings are summarized in (Table 4).

## 6. Pivotal Trials and Ongoing Clinical Development of Odronextamab

The comprehensive ELM clinical development program provides robust validation of odronextamab across B-cell non-Hodgkin lymphomas.

The ELM-1 trial was a pivotal multicenter, phase I study investigating the efficacy and safety of odronextamab in 145 patients with heavily pretreated R/R B-NHL, including individuals refractory to multiple prior therapies and those previously treated with CAR T-cell therapy. The study reported an ORR) of 51%, with particularly high activity in FL (ORR 91%; CR 72%). Clinically meaningful CRs were also observed in patients with DLBCL who had not received prior CAR T-cell therapy. The safety profile was manageable and characterized primarily by CRS, which occurred in 48.3% of patients and was exclusively grade 1–2. Neurologic adverse events were reported in approximately 50% of patients, most commonly headache, anxiety, and encephalopathy, and were generally low grade; no cases of ICANS were identified. Infections occurred in approximately 50% of patients, with grade ≥ 3 infections in 20%, and included COVID-19, pneumonia, and device-related infections; rare fatal events included COVID-19 pneumonia [92]. In a dedicated expansion cohort of patients with DLBCL progressing after CAR T-cell therapy, odronextamab achieved an ORR of 48.3% and a CR rate of 31.7%, with a median DoR of 14.8 months. Notably, no severe CRS or ICANS events were observed in this high-risk population, supporting the safety of odronextamab in the post-CAR T setting [93].

Results from the phase II ELM-2 study further confirmed durable clinical activity in heavily pretreated R/R DLBCL. In an efficacy analysis of 127 patients (median follow-up approximately 27 months), odronextamab achieved an ORR of 52% and a CR rate of 31.5%, with a median duration of CR of 17.9 months and sustained responses over time [94,95]. Early molecular markers also proved predictive, as undetectable minimal residual disease by the fourth treatment cycle correlated with improved PFS. The safety profile in ELM-2 was characterized by frequent but predominantly low-grade CRS, with grade ≥ 3 events reported in a small proportion of patients. Implementation of optimized step-up dosing effectively mitigated neurotoxicity, resulting in zero reported cases of ICANS [95]. Neurologic adverse events occurred in approximately 43% of patients and were mostly grade ≤ 2. Infections were common (approximately 65%), with COVID-19 representing the most frequent infection and the leading cause of grade 5 infectious events. Despite their frequency, infections were generally manageable and were associated with B-cell depletion and hypogammaglobulinemia [94]. Hematologic toxicity, including neutropenia and thrombocytopenia, was also observed. Patient-reported outcomes indicated that quality of life was maintained or improved throughout treatment [94,95]. Pharmacokinetic and pharmacodynamic data from the ELM-1 and ELM-2 trials supported a weight-independent dosing regimen for odronextamab in DLBCL. The drug is administered intravenously using a step-up schedule during cycle 1 (0.2 mg on day 1, 0.5 mg on day 2, 2 mg on days 8 and 9, and 10 mg on days 15 and 16), followed by 160 mg weekly during cycles 2–4 and 320 mg every 2 weeks thereafter, with the option to extend dosing to every 4 weeks in patients achieving sustained CR. Infusions are administered over several hours with gradual rate escalation, particularly during early doses, to mitigate infusion-related reactions. Premedication with corticosteroids, antihistamines, and antipyretics is required during the initial cycles to reduce the risk of CRS [96].

Odronextamab has also demonstrated substantial activity in R/R FL across phase I/II studies, with CR rates approaching 70% and durable remissions. The safety profile in FL was consistent with that observed in DLBCL, with CRS occurring in approximately 56–57% of patients and predominantly during step-up dosing, and hematologic toxicity such as neutropenia, representing the most common grade ≥ 3 adverse event. Infections were frequent, occurring in up to 80% of patients, and included pneumonia, viral bronchitis, pseudomonal pneumonia, COVID-19 pneumonia, and opportunistic infections such as Pneumocystis jirovecii pneumonia, cytomegalovirus, and systemic fungal infections. Grade ≥ 3 infections occurred in approximately 30% of patients, and rare fatal infections included pneumonia, progressive multifocal leukoencephalopathy (PML), pseudomonal pneumonia, and COVID-19 pneumonia with systemic mycosis. ICANS was rare, with only isolated low-grade events reported [92,97,98]. Based on these findings, several phase III trials are currently evaluating odronextamab-based combinations in various other settings.

The OLYMPIA-5 trial is investigating odronextamab plus lenalidomide versus R^2^ in patients with R/R FL and marginal zone lymphoma (MZL) [99,100]. Efficacy signals were encouraging, with objective response rates of 83.3% and 88.5% across dose levels and CR rates of 65–67%, while DoR had not been reached [100]. The randomized Part 2 aims to confirm improvements in PFS, OS, and response depth compared with R2. In the safety lead-in phase, the combination demonstrated a manageable safety profile with no dose-limiting toxicities and predominantly grade 1–2 CRS. Hematologic toxicity, particularly neutropenia, was common, while infections occurred in approximately 67–77% of patients, with grade ≥ 3 infections in up to one-third of cases. A single grade 3 ICANS event was reported [100].

In the untreated FL setting, the OLYMPIA-1 trial is evaluating odronextamab monotherapy versus the investigator’s choice of chemoimmunotherapy, incorporating step-up dosing over six 21-day cycles followed by maintenance in responders. Early safety data indicate that CRS events are low-grade. No ICANS or tumor lysis syndrome was reported, and only isolated grade ≥ 3 infections were observed [101].

The OLYMPIA-2 study, which also focuses in untreated FL cases, is evaluating odronextamab in combination with chemotherapy with or without maintenance versus standard rituximab-based chemoimmunotherapy. Early results from the dose-escalation and optimization phase demonstrated high activity, with objective response rates approaching 90–100% and CR rates up to 86%, alongside a manageable safety profile with predominantly low-grade CRS. Hematologic toxicity, including neutropenia and anemia, represents the most common high-grade adverse events. Infections occurred in up to 77% of patients, with grade ≥ 3 infections reported in up to 44%, occasionally requiring prolonged hospitalization. CRS events were limited to grade 1–2, and no ICANS events were reported [102].

Beyond these approaches, novel immunotherapy combinations are under investigation. Odronextamab is being evaluated in combination with cemiplimab and REGN5837 within the phase I ATHENA-1 trial [103,104,105], as well as in combination with cemiplimab in R/R CD20-positive B-NHL [106].

Overall, odronextamab demonstrates a consistent safety profile characterized by early-onset, predominantly low-grade CRS, infrequent neurotoxicity, and a high incidence of infections. These infections are predominantly respiratory (COVID-19, pneumonia), whilesepsis and fungal infections represent rare but clinically significant severe events. The key clinical studies of odronextamab across different disease settings are summarized in (Table 5).

## 7. Conclusions

CD20 × CD3 BsAbs have emerged as an effective therapeutic class in B-cell non-Hodgkin lymphomas, particularly in the R/R setting, although their use in the frontline setting is currently actively evaluated. Their off-the-shelf availability enables rapid treatment initiation without the need for patient-specific manufacturing, while administration in standard clinical settings—including outpatient care—facilitates broader accessibility compared with cellular T-cell-engaging therapies [16,114].

Despite differences in molecular design, route of administration, and dosing strategies, currently available BsAbs demonstrate consistent clinical activity across both aggressive and indolent lymphomas, including high-risk populations and patients previously treated with CAR T-cell therapy [15,16,31,42,56,57,64,69,70,73,92,96].

On the other hand, the four approved CD20 × CD3 BsAbs show clinically meaningful differences in safety and administration. CRS is a consistent class effect, typically occurring early during treatment and being predominantly low grade, although higher rates have been reported with glofitamab, particularly in combination regimens [55,61,62]. Mosunetuzumab is generally associated with a more favorable CRS profile, which will be further improved with the subcutaneous formulation [28,29], while in subcutaneous epcoritamab, CRS shows predictable timing and manageable kinetics [78,79,80,81]. Neurologic toxicity, including ICANS, is uncommon and typically low grade [55,78,94]. Differences in administration may also influence clinical use, with subcutaneous delivery offering potential advantages for outpatient management [78,81].

Infection risk differs across agents. Lower rates of severe infections have been reported with mosunetuzumab [28,29], whereas glofitamab and epcoritamab, especially in combination settings, are associated with increased infections, including COVID-19-related events and opportunistic pathogens [61,62,81,87]. Odronextamab appears to be associated with a higher overall incidence of infections, including opportunistic infections, likely related to deeper B-cell depletion and hypogammaglobulinemia [94,97,98]. Hematologic toxicity, particularly neutropenia, is observed across all agents and is more frequent in combination regimens, contributing to infection risk [55,81,94]. In general, infections are increasingly recognized as a major toxicity of CD20 × CD3 BsAbs, beyond CRS and ICANS. In a meta-analysis of 27 lymphoma studies with BsAbs [115], all-grade infections occurred in 44% of patients, with product-specific rates ranging from 39% with epcoritamab to 59% with odronextamab; the highest pooled estimate was reported with odronextamab, although this should be interpreted in the context of cross-trial heterogeneity and follow-up duration. Grade ≥ 3 and fatal infections occurred in 20% and 3% of patients, respectively, and fatal microbiologically documented events were predominantly viral, largely driven by SARS-CoV-2. This pattern likely reflects sustained B-cell depletion, hypogammaglobulinemia, prior treatment burden and impaired immune reconstitution, and supports CAR T-like supportive-care strategies, including serial IgG monitoring and early consideration of immunoglobulin replacement in selected high-risk patients (e.g., when IgG is below 400 mg/dL) [116].

The above safety data should not be interpreted as allowing direct cross-trial comparisons between agents, as the available studies differ substantially in terms of patient populations, lymphoma subtypes, treatment settings, prior lines of therapy, combination partners, follow-up duration, and calendar period of recruitment, including variable overlap with the COVID-19 pandemic.

Ongoing clinical development is rapidly expanding the role of BsAbs into earlier lines of therapy, both as chemotherapy-free approaches and in combination with established regimens. However, key challenges remain, including optimal patient selection, treatment sequencing, and the impact of emerging resistance mechanisms on long-term outcomes. These mechanisms are not fully elucidated; however, several explanations have been proposed. Tumor-intrinsic mechanisms, such as loss or downregulation of CD20 expression, may reduce target engagement and contribute to treatment failure [26,117]. In parallel, alterations in interferon-γ-dependent signaling pathways have been implicated in impaired immune-mediated cytotoxicity and tumor immune escape [26]. Moreover, sustained T-cell engagement may lead to progressive T-cell dysfunction, with features consistent with exhaustion, including reduced proliferative capacity and diminished effector function [15,26]. In addition, the immunosuppressive tumor microenvironment—characterized by regulatory T cells, inhibitory cytokines, and checkpoint signaling—may further limit the efficacy of T-cell redirection strategies [15,114,118]. These mechanisms are likely to have important implications for treatment sequencing, depth and durability of response, and long-term disease control. Future research is therefore focused on identifying predictive biomarkers and developing rational combination strategies to overcome resistance and optimize clinical outcomes. These observations may help explain variability in clinical response and support the development of biomarker-driven treatment strategies.

Finally, the positioning of BsAbs relative to CAR T-cell therapy—the other major pillar of T-cell-engaging immunotherapy—continues to evolve as both modalities are increasingly used in overlapping clinical settings. To date, the largest body of evidence concerns the use of BsAbs after CAR T-cell therapy failure, where several studies have shown meaningful clinical activity in this difficult-to-treat population [119]. Notably, responses to BsAbs in CAR T-cell-exposed patients appear to be substantial, and in some reports approach those observed in CAR T-cell-naïve patients [61], suggesting that prior cellular therapy does not necessarily preclude effective T-cell redirection with BsAbs. Conversely, the available experience with CAR T-cell therapy after BsAb exposure remains more limited and is derived mainly from real-world datasets; nevertheless, the reported outcomes are encouraging and indicate that CAR T-cell therapy may retain activity in patients previously treated with BsAbs [119].

Overall, BsAbs represent a major advancement in the therapeutic landscape of B-cell lymphomas, with their ultimate clinical value likely to be defined by optimal integration across treatment lines, rational sequencing with cellular therapies, and careful balancing of efficacy, safety, and accessibility.

## Figures and Tables

**Table 1:** Current regulatory approvals of CD20 × CD3 bispecific antibodies in B-cell lymphomas [17,18,19,20,21,22,23].

Agent	FDA Approval	EMA Approval
Mosunetuzumab [17,18]	R/R FL (≥2 prior lines), monotherapy	R/R FL (≥2 prior lines), monotherapy
Glofitamab [19,20]	R/R DLBCL (≥2 prior lines), monotherapy	• R/R DLBCL (≥2 prior lines), monotherapy • R/R DLBCL: in combination with GemOx for transplant-ineligible patients (≥1 prior line)
Epcoritamab [21,22]	• R/R DLBCL and FL (≥2 prior lines), monotherapy • R/R FL in combination with R^2^	R/R DLBCL and FL (≥2 prior lines), monotherapy
Odronextamab [23]	Not approved	R/R FL and DLBCL (≥2 prior lines), monotherapy

R/R: relapsed/refractory, FL: follicular lymphoma, DLBCL: diffuse large B-cell lymphoma, GemOx: gemcitabone-oxaliplatine, R^2^: lenalidomide-rituximab.

**Table 2:** Key Clinical Trial Characteristics, Efficacy Outcomes, and Safety Profile of Mosunetuzumab in B-Cell Non-Hodgkin Lymphomas [28,29,31,32,33,34,35,36,41,42,43,44,45,46,47,48,49,50,51,52].

NCT Identifier	Phase	Population	Regimen	Key Efficacy Results	Adverse Events
NCT02500407 (GO29781) [28,29,41]	II	R/R FL ≥ 3 L	Monotherapy	ORR 78.0%, CR 60.0%; median DoR 35.9 mo; OS 82.4%.	CRS: 27.4% (grade ≥ 3: 1.0%); ICANS: NR; Neutropenia: 28.4% (grade ≥ 3: 25.4%); Febrile neutropenia: 3.6% (grade ≥ 3: 3.6%); Thrombocytopenia: 2.5% (grade ≥ 3: 2.0%); Pneumonia: 4.6% (grade ≥ 3: 2.5%); UTI: 7.6% (grade ≥ 3: 2.5%).
NCT03671018 (GO40516) [42,43]	Ib/II	R/R LBCL ≥ 2 L	Mosunetuzumab + polatuzumab vedotin	ORR 59.2%, CR 45.9%; median DoR not reached.	CRS: 16.7% (grade ≥ 3: 2.5%); ICANS: 4.6% (grade ≥ 3: 1.6%); Neutropenia: 35.0% (grade ≥ 3: 25.0%); Febrile neutropenia: 0.0%; Infections: 39.2% (grade ≥ 3: 8.3%; pneumonia: 9.2%; COVID-19: 4.2%).
NCT05171647 (SUNMO) [31,44]	III	R/R LBCL transplant-ineligible ≥ 2 L	Mosunetuzumab + polatuzumab vs. R-GemOx	ORR 70.0% vs. 40.0%; CR 51.4% vs. 24.3%; median PFS 11.5 vs. 3.8 mo (HR 0.41); OS 18.7 vs. 13.6 mo.	CRS: 26.0% (grade ≥ 3: 0.7%) vs. 0.0%; ICANS: 0.0% vs. 0.0%; Neutropenia: 46.0% (grade ≥ 3: 33.0%) vs. 55.0% (grade ≥ 3: 31.0%); Febrile neutropenia: 2.2% vs. 3.1%; Thrombocytopenia: 8.9% (grade ≥ 3: 2.2%) vs. 66.0% (grade ≥ 3: 36.0%); Infections: 51.0% vs. 31.0% (grade ≥ 3: 16.0% vs. 14.0%; pneumonia: 5.2% vs. 0.0%; COVID-19: 15.0% vs. 3.1%).
NCT03677154 [32,45]	II	Untreated DLBCL	Monotherapy vs. Mosunetuzumab + polatuzumab	ORR ~55–60%, CR ~40–45%; 12-mo PFS ~35–40%.	CRS: 26.0% (grade ≥ 3: 0.0%); ICANS: 0.0%; Neutropenia: 15.0% (grade ≥ 3: 13.0%); Febrile neutropenia: 1.9%; Infections: 22.0% (grade ≥ 3: 21.0%; COVID-19: 4.0%).
NCT03677141 [33,46]	II	Untreated DLBCL	Pola-M-CHP vs. Pola-R-CHP	ORR 75.0% vs. 86.4%; CR 72.5% vs. 73.1%; 12-mo PFS 70.8% vs. 81.8%; 12-mo DoR 75.0% vs. 85.7%.	CRS: 68.4% (grade ≥ 3: 2.6%) vs. 0.0%; ICANS: 0.0% vs. 0.0%; Neutropenia: 63.2% (grade ≥ 3: 63.2%) vs. 54.5% (grade ≥ 3: 40.9%); Febrile neutropenia: 13.2% (grade ≥ 3: 13.2%) vs. 9.1% (grade ≥ 3: 9.1%); Thrombocytopenia: 15.8% (grade ≥ 3: 7.9%) vs. 4.5% (grade ≥ 3: 0.0%); Infections: 10.5% vs. 4.5% (grade ≥ 3: 7.9% vs. 4.5%; pneumonia: 0.0% vs. 4.5%).
NCT05207670 (MorningSun) [34,47]	II	Untreated elderly/unfit DLBCL	Mosunetuzumab SC	ORR 73.5%, CR 59.2%; 12-mo PFS 68.8%; 12-mo DoR 84.6%.	CRS: 12.2% (grade ≥ 3: 0.0%); ICANS: 0.0%; Neutropenia: 15.0% (grade ≥ 3: NR); Infections: 46.9% (grade ≥ 3: 16.3%).
NCT04712097 (CELESTIMO) [35,48]	III	R/R FL (≥1 prior line), early relapse	Mosunetuzumab + lenalidomide vs. R^2^	ORR 96.3%, CR 87.0%.	CRS: 27.8% (grade ≥ 3: 1.9%); ICANS: NR; Neutropenia: 40.7% (grade ≥ 3: 33.3%); Febrile neutropenia: 3.7%; Infections: 57.4% (COVID-19: 20.4%).
NCT05207670 (MorningSun) [36,47]	II	Untreated FL (high tumor burden)	Mosunetuzumab SC	ORR 87.4%, CR 64.1%; 12-mo PFS 85.5%; OS 91.9%.	CRS: 48.5% (grade ≥ 3: 0.0%); ICANS: NR; Neutropenia: 40.7% (grade ≥ 3: 33.3%); Infections: 63.4% (grade ≥ 3: 11.9%; pneumonia: 11.9%; COVID-19: 17.8%).
NCT06284122 (MorningLyte) [49]	III	Untreated FL	Mosunetuzumab + lenalidomide vs. chemoimmunotherapy	No results yet.	No results yet.
NCT05849857 (MERLIN) [50]	II	FL (POD24), early relapse	Mosunetuzumab SC	No results yet.	No results yet.
NCT05994235 [51]	II	Untreated FL	Mosunetuzumab + tazemetostat	No results yet.	No results yet.
NCT06249191 [52]	Ib/II	HGBCL/DLBCL	Mosunetuzumab + DA-EPOCH	No results yet.	No results yet.

Abbreviations: R/R, relapsed/refractory; FL, follicular lymphoma; DLBCL, diffuse large B-cell lymphoma; LBCL, large B-cell lymphoma; HGBCL, high-grade B-cell lymphoma; ORR, overall response rate; CR, complete response; DoR, duration of response; PFS, progression-free survival; OS, overall survival; HR, hazard ratio; mo, months; R, rituximab; R^2^, lenalidomide plus rituximab; GemOx, gemcitabine plus oxaliplatin; SC, subcutaneous; POD24, progression of disease within 24 months; DA-EPOCH, dose-adjusted etoposide, prednisone, vincristine, cyclophosphamide, and doxorubicin; CRS, cytokine release syndrome; ICANS, immune effector cell-associated neurotoxicity syndrome; UTI, urinary tract infection.

**Table 3:** Key Clinical Trial Characteristics, Efficacy Outcomes, and Safety Profile of Glofitamab in B-Cell Non-Hodgkin Lymphomas [53,54,55,56,59,61,62,63,64,65,66,67,68,70,71,72,73,74,75].

NCT	Phase	Population	Regimen	Key Results	Adverse Events
NCT03075696 (ELM/NP30179) [53,54]	I/Ib	R/R MCL (≥2 L)	Monotherapy	ORR 85.0%; CR 78.3%; Median DoR 15.4 mo; Median DoR (updated) 16.2 mo; Median PFS 16.8 mo	CRS: 70.0% (grade ≥ 3: 11.7%); ICANS: NR; Neutropenia: 38.3% (grade ≥ 3: 23.3%); Pneumonia: 11.7%; COVID-19: 31.7%.
NCT03075696 (ELM/NP30179) [53,55,56,57]	I/II	R/R LBCL (≥2 L)	Monotherapy	ORR 54–56%; CR 57.0%; Median DoR 18.4 mo; Median PFS 4.9 mo	CRS: 63.0% (grade ≥ 3: 4.0%); ICANS: 8.0% (grade ≥ 3: 3.0%); Neutropenia: 27.3% (grade ≥ 3: 27.0%); Febrile neutropenia: 3.0% (grade ≥ 3: 3.0%); Infections: 38.0% (grade ≥ 3: 15.0%; COVID-19: 9.0% (grade ≥ 3: 6.0%).
NCT04703686 (LYSA) [59,71]	II	R/R DLBCL after CAR-T failure	Monotherapy	ORR 76.1%; CR 45.7%; Median DoR 19.7 mo; Median PFS 3.8 mo	CRS: 13.0% (grade ≥ 3: 0.0%); ICANS: 2.2% (grade ≥ 3: 0.0%); Neutropenia: NR (grade ≥ 3: 34.8%); Infections: (COVID-19: 13.0%).
NCT04408638 (STARGLO) [61,62,72]	III	R/R DLBCL (ASCT-ineligible) (≥2 L)	Glofitamab + GemOx vs. R-GemOx	CR 50.3% vs. 22.2%; Median DoR NR vs. 24.2 mo; Median PFS 13.8 mo vs. 3.6 mo	CRS: 44.4% (grade ≥ 3: 2.0%) vs. 0.0%; ICANS: 2.0% (grade ≥ 3: 1.0%) vs. 0.0%; Neutropenia: 42.0% (grade ≥ 3: 3.0%) vs. 31.0% (grade ≥ 3: 1.0%); Febrile neutropenia: 3.0% (grade ≥ 3: NR) vs. 1.0% (grade ≥ 3: NR); Thrombocytopenia: 48.0% vs. 48.0%; Serious infection: 26.0% vs. 13.0%; pneumonia grade 5: 3.0% vs. 1.0%; COVID-19: 18.0% vs. 9.0%.
NCT03533283 [63,64,65,66]	I/II	R/R LBCL (incl. HGBCL & post-CAR-T) (≥2 L)	Glofitamab + Polatuzumab vedotin	ORR 78.3%; CR 59.7%; Median DoR 37.8 mo; Median PFS 12.3 mo	CRS: 43.4% (grade ≥ 3: 1.6%); ICANS: 3.1% (grade ≥ 3: 0.0%); Neutropenia: 41.9% (grade ≥ 3: 32.6%); Thrombocytopenia: grade ≥ 3: 8.5%; Infections: 60.5% (grade ≥ 3: 23.3%; pneumonia: 10.9% [grade ≥ 3: 4.7%]; COVID-19: 23.3% [grade ≥ 3: 9.4%]).
NCT04914741 (COALITION) [68,73]	I/II	Untreated LBCL—High risk (Frontline)	Glofitamab + R-CHOP vs. Glofitamab + Pola-R-CHP	ORR 100%; CR 98.0%; Median DoR NR; Median PFS NR	CRS: 21.0% (grade ≥ 3: 0.0%); ICANS: 0.0%; Neutropenia: NR (grade ≥ 3: 55.0%); Infections: (grade ≥ 3: 13.0%).
NCT04980222 [70,74]	I/II	Untreated DLBCL (Frontline)	Glofitamab + R-CHOP	ORR 93.3%; CR 80.0%; Median DoR NR; Median PFS NR	CRS: 20.8% (grade ≥ 3: 0.0%); ICANS: 0.0%; Neutropenia: 54.2% (grade ≥ 3: 45.8%); Thrombocytopenia: 12.5% (grade ≥ 3: 4.2%); Infections: (pneumonia: NR; COVID-19: 16.7%).
NCT06047080 (SKYGLO) [67,75]	III	Untreated LBCL (Frontline)	Glofitamab + Pola-R-CHP vs. Pola-R-CHP	No results yet.	No results yet.

Abbreviations: R/R, relapsed/refractory; DLBCL, diffuse large B-cell lymphoma; LBCL, large B-cell lymphoma; MCL, mantle cell lymphoma; ORR, overall response rate; CR, complete response; DoR, duration of response; PFS, progression-free survival; OS, overall survival; mo, months; R^2^, lenalidomide plus rituximab; GemOx, gemcitabine plus oxaliplatin; Pola, polatuzumab vedotin; ASCT, autologous stem cell transplantation; CRS, cytokine release syndrome; ICANS, immune effector cell-associated neurotoxicity syndrome.

**Table 4:** Key Clinical Trial Characteristics, Efficacy Outcomes, and Safety Profile of Epcoritamab in B-Cell Non-Hodgkin Lymphomas [21,22,76,77,78,79,80,81,82,83,84,85,86,87,88,89,90,91].

NCT	Phase	Population	Regimen	Key Efficacy Outcomes	Adverse Events
NCT03625037 (EPCORE NHL-1) [76,78,79,82]	I/II	R/R B-NHL (DLBCL, FL), ≥2 prior lines	Epcoritamab (monotherapy)	**Dose escalation:** DLBCL: ORR 68%, CR 45% (12–60 mg); FL: ORR 90%, CR 50%. **Dose expansion:** DLBCL: ORR 63%, CR 39%, median DOR 12.0 months FL: ORR 82%, CR 63%.	**DLBCL:** CRS: 51.0% (grade ≥ 3: 3.2%); ICANS: 6.4% (grade ≥ 3: 0.6%); Neutropenia: 23.6% (grade ≥ 3: 16.6%); Febrile neutropenia: 2.5% (grade ≥ 3: 2.5%); Thrombocytopenia: 12.1% (grade ≥ 3: 5.0%); Infections: (grade ≥ 3: 25.5%; pneumonia: 8.3%; COVID-19: 19.1% [grade ≥ 3: 8.3%]). **FL:** CRS: 60.9% (grade ≥ 3: 1.9%); ICANS: NR; Neutropenia: 64.8% (grade ≥ 3: 54.6%); Febrile neutropenia: NR; Thrombocytopenia: 21.3% (grade ≥ 3: 8.8%); Pneumonia: 21.3% (grade ≥ 3: 12.0%); COVID-19: 39.3% (grade ≥ 3: 24.1%); UTI: 17.6% (grade ≥ 3: 2.8%).
NCT04663347 (EPCORE NHL-2) [77,83,84,85,86]	I/II	DLBCL & FL (frontline and R/R)	Epcoritamab + combinations	**DLBCL (R/R):** GemOx: ORR 85%, CR 61%; R-DHAX/C: CR 65–70%; R-ICE: CR 60–65%. **DLBCL (frontline):** R-CHOP: ORR ~100%, CR 85–87%; R-mini-CHOP: ORR 89%, CR 86%. **FL (R/R):** R^2^: ORR 96%, CR 88%; Lenalidomide (POD24): active (mature data NR). **FL (frontline):** BR: CR ~95% (early data). **FL (maintenance/novel strategies):** Epcoritamab (post-1 L/2 L SOC): mDOR NR, mDOCR NR, mOS NR; 30-month DOCR 88%, OS 84%. R^2^: 33-month DOR 89%, DOCR 93%; 36-month PFS 86%, OS 88%.	**DLBCL (R/R)—GemOx:** CRS: 52.4% (grade ≥ 3: 1.0%); ICANS: 2.9% (grade ≥ 3: 1.0%); Neutropenia: 65.0% (grade ≥ 3: 57.3%); Febrile neutropenia: 7.0% (grade ≥ 3: 7.0%); Thrombocytopenia: 72.8% (grade ≥ 3: 59.2%); Infections: 71.8% (grade ≥ 3: 29.1%; pneumonia: 10.0%; COVID-19: 13.0%; UTI: 10.0%). **FL (R/R)—R^2^:** CRS: 50.9% (grade ≥ 3: 1.9%); ICANS: NR; Neutropenia: 64.8% (grade ≥ 3: 54.6%); Febrile neutropenia: 3.0%; Thrombocytopenia: 21.3% (grade ≥ 3: 5.6%); Infections: 71.8% (grade ≥ 3: 29.1%; pneumonia: 21.3%; COVID-19: 59.3%; UTI: 17.6%).
NCT05409066 (EPCORE FL-1) [86,87]	III	R/R FL ≥ 1 L	Epcoritamab + R^2^ vs. R^2^	ORR 95% vs. 79%; PFS NR (HR~0.2)	CRS: 35.0% (grade ≥ 3: 0.0%) vs. 0.0%; ICANS: NR; Neutropenia: 74.0% (grade ≥ 3: 69.0%) vs. 52.0% (grade ≥ 3: 42.0%); Febrile neutropenia: 6.0% vs. 3.0%; Thrombocytopenia: 28.0% (grade ≥ 3: 9.0%) vs. 18.0% (grade ≥ 3: 6.0%); Infections: 77.0% vs. 53.0% (grade ≥ 3: 33.0% vs. 16.0%; pneumonia: 19.0% vs. 8.0%; COVID-19: 22.0% vs. 13.0%).
NCT04628494 (EPCORE DLBCL-1) [88,89]	III	R/R DLBCL ≥ 2 L	Epcoritamab vs. SOC	PFS HR 0.74; improved CR, DoR, TTNT; OS NS	No published data yet
NCT05578976 (EPCORE DLBCL-2) [90]	III	Previously untreated DLBCL	Epcoritamab + R-CHOP vs. R-CHOP	No results yet.	No results yet.
NCT06508658 (EPCORE DLBCL-4) [91]	III	R/R DLBCL, ASCT-ineligible	Epcoritamab + lenalidomide vs. SOC	No results yet.	No results yet.

Abbreviations: R/R, relapsed/refractory; DLBCL, diffuse large B-cell lymphoma; LBCL, large B-cell lymphoma; FL, follicular lymphoma; CRS, cytokine release syndrome; ICANS, immune effector cell-associated neurotoxicity syndrome; ORR, overall response rate; CR, complete response; DoR, duration of response; PFS, progression-free survival; OS, overall survival; TTNT, time to next treatment; HR, hazard ratio; NR, not reported (either not described in the study or not yet available, particularly for early-phase trials); R^2^, rituximab plus lenalidomide; ASCT, autologous stem cell transplantation; GemOx, gemcitabine plus oxaliplatin; SOC, standard of care; UTI, urinary tract infection.

**Table 5:** Key Clinical Trial Characteristics, Efficacy Outcomes, and Safety Profile of Odronextamab in B-Cell Non-Hodgkin Lymphomas [92,93,94,95,96,97,98,99,100,101,102,103,104,105,106,107,108,109,110,111,112,113].

NCT	Phase	Population	Regimen	Key Efficacy Results	Adverse Events
NCT03888105 (ELM-2) [94,95,97,98,107]	II	R/R DLBCL & FL ≥ 3 L	Monotherapy	**DLBCL**: ORR 52.0%; CR 31.5%; Median DoR 17.9 mo (updated 10.2 mo); Median PFS NR. **FL:** ORR 80.0%; CR 73.0%; Median DoR 25.1 mo; Median PFS 20.7 mo.	**DLBCL:** CRS: 55.1% (grade ≥ 3: 4.7%); ICANS: 0.0%; Neutropenia: 30.7% (grade ≥ 3: 26.0%); Thrombocytopenia: 18.9% (grade ≥ 3: 15.0%); Infections: 64.0% (grade ≥ 3: 38.6%; COVID-19: 16.0%). **FL:** CRS: 55.0% (grade ≥ 3: 1.7%); ICANS: NR; Infections: 81.3% (grade ≥ 3: 46.1%; COVID-19: 39.8%).
NCT02290951 (ELM-1) [92,93,108]	I	R/R B-NHL (incl. post-CAR-T) ≥ 3 L	Monotherapy	**DLBCL:** ORR 48.3%, CR 31.7%; median DoR 14.8 mo; median PFS 4.8 mo; OS NR.	CRS: 61.0% (grade ≥ 3: 10.0%); ICANS: 12.0% (grade ≥ 3: 3.0%); Neutropenia: 25.0% (grade ≥ 3: 19.0%); Thrombocytopenia: 28.0% (grade ≥ 3: 14.0%); Infections: 49.0% (grade ≥ 3: 23.0%; pneumonia: 11.0%; COVID-19: 1.0%; fungal: 3.0%; UTI: 1.0%).
NCT06091254 (OLYMPIA-1) [101,109]	III	Untreated FL (frontline)	Odronextamab vs. chemoimmunotherapy	Early safety lead-in: high CR rates (~100% at week 12); efficacy immature.	CRS: 30.8% (grade ≥ 3: 0.0%); ICANS: 0.0%; Infections: (grade ≥ 3: 7.7%).
NCT06097364 (OLYMPIA-2) [102,110]	III	Untreated FL (frontline)	Odronextamab + CHOP/CVP	ORR 88.9–100%; CR 78–86% (dose-dependent).	CRS: 22.2–58.3% (grade ≥ 3: 0.0%); ICANS: 0.0%; Neutropenia: 55.6–83.3% (grade ≥ 3: 55.6–83.3%); Infections: 66.7–77.8% (grade ≥ 3: 16.7–44.4%).
NCT06149286 (OLYMPIA-5) [99,100,111]	III	R/R FL & MZL ≥ 2 L	Odronextamab + lenalidomide vs. R^2^	ORR 83–88%; CR 65–67%; median DoR not reached.	CRS: 53.8% (grade ≥ 3: 0.0%); ICANS: 3.8% (grade ≥ 3: 3.8%); Neutropenia: 66.7–76.9% (grade ≥ 3: 66.7–76.9%); Infections: 66.7–76.9% (grade ≥ 3: 23.1–33.3%).
NCT02651662 (CELLO-1) [106,112]	I/II	R/R B-NHL ≥ 2 L	Odronextamab + cemiplimab	No results yet.	No results yet.
NCT05685173 (ATHENA-1) [104,113]	I/II	R/R aggressive B-NHL ≥ 2 L	Odronextamab + REGN5837 (CD22 × CD28)	No results yet.	No results yet.

Abbreviations: R/R, relapsed/refractory; B-NHL, B-cell non-Hodgkin lymphoma; DLBCL, diffuse large B-cell lymphoma; FL, follicular lymphoma; MZL, marginal zone lymphoma; CRS, cytokine release syndrome; ICANS, immune effector cell-associated neurotoxicity syndrome; ORR, overall response rate; CR, complete response; DoR, duration of response; PFS, progression-free survival; OS, overall survival; NR, not reported (includes data not described in the study or not yet available for early-phase trials); Lena, lenalidomide; UTI, urinary tract infection.

## Data Availability

Data sharing is not applicable to this article as no new data were created or analyzed in this study.

## Abbreviations

The following abbreviations are used in this manuscript:

ASCT	autologous stem cell transplantation
B-NHL	B-cell non-Hodgkin lymphoma
CR	complete response
CRS	cytokine release syndrome
DA-EPOCH	dose-adjusted etoposide, prednisone, vincristine, cyclophosphamide, and doxorubicin
DLBCL	diffuse large B-cell lymphoma
DoR	duration of response
FL	follicular lymphoma
GemOx	gemcitabine plus oxaliplatin
HGBCL	high-grade B-cell lymphoma
HR	hazard ratio
ICANS	immune effector cell-associated neurotoxicity syndrome
IRC	independent review committee
LBCL	large B-cell lymphoma
Lena	lenalidomide
MCL	mantle cell lymphoma
mo	months
MZL	marginal zone lymphoma
NHL	non-Hodgkin lymphoma
NR	not reported
ORR	overall response rate
OS	overall survival
PFS	progression-free survival
POD24	progression of disease within 24 months
Pola	polatuzumab vedotin
R	rituximab
R/R	relapsed/refractory
R^2^	lenalidomide plus rituximab
sc	subcutaneous
SOC	standard of care
UTI	urinary tract infection
